# Wayfinding behavioral patterns of seniors with dementia: two exploratory case studies

**DOI:** 10.3389/frdem.2025.1524425

**Published:** 2025-03-10

**Authors:** Leonie van Buuren, Daantje Derks, Masi Mohammadi, Bernard Colenbrander

**Affiliations:** ^1^Department of Built Environment, Eindhoven University of Technology, Eindhoven, Netherlands; ^2^Department of Psychology, Education, and Child Studies, Erasmus University Rotterdam, Rotterdam, Netherlands

**Keywords:** spatial orientation, wayfinding, nursing home, fly-on-the-wall observation, dementia, circulation

## Abstract

**Introduction:**

While wayfinding is vital for quality of life, it is also a declining skill for people with dementia. Understanding wayfinding behavioral patterns of people with dementia helps to improve the nursing home corridor designs to facilitate autonomously conducting activities of daily life. However, a comprehensive image of these patterns is lacking.

**Methods:**

An empirical qualitative study was conducted, studying seven wayfinding behavioral patterns of people with advanced dementia (*n* = 8) in two nursing home corridors where they live, using fly-on-the-wall observation.

**Results:**

The data show that the most frequent wayfinding behavioral patterns observed were “movements” followed by “looking at”, “stops on the route”, and “verbal navigational cues”.

**Discussion:**

These behaviors occurred often at crossroads; i.e., places in which participants should make a decision concerning continuing their route. Spatially, these places have high-visibility values and many things to see for people with dementia. Contradictory, these places might cause more confusion for people with dementia. Therefore, special attention should be paid to the design of these spaces.

## 1 Introduction

Understanding wayfinding behavioral patterns of people with dementia in nursing home corridors is critical to optimizing these spaces' spatial designs (Kuliga et al., 2021). Wayfinding is essential for autonomously conducting activities of daily life and hence maintaining—or even improving—quality of life. This is especially the case for people with dementia (Andersen et al., 2004; Frierson and Jacoby, 2008; Marquardt, 2011) because cognitive processes related to wayfinding decline due to their dementia (Pai and Jacobs, 2004; Reisberg et al., 1982). Also, feelings of being lost are associated with the experience of stress (e.g., Delgrange et al., 2020) which cumulatively negatively impacts the quality of life of people with dementia.

Wayfinding happens all day since you move from one space to another to conduct certain activities. The physical environment influences this wayfinding behavior, particularly for people with dementia, due to their dependency on this physical environment (Lawton and Simon, 1968). People with advanced stages of dementia are, at a particular moment, unable to live independently at home and have to move to a nursing home (den Draak et al., 2016). Research shows that the current designs of Dutch nursing homes could be improved to facilitate wayfinding (Davis and Weisbeck, 2016; van Buuren and Mohammadi, 2022). To improve these designs, research into wayfinding behaviors in these settings can contribute insights.

Reaching your destination within a building means that you have to move through space(s). Where wayfinding is the (environmental) psychological perspective of reaching a destination, the design of the circulation space and the composition of spaces is the architectural perspective (Passini, 1996).

From the environmental psychological perspective, spatial orientation is defined as mentally determining your position in a spatial setting, while dynamic cognitive processes to reach a destination are called wayfinding (Passini, 1984). In literature, multiple theoretical frameworks on wayfinding exist. In this current study, wayfinding as spatial-problem solving process of Passini (1984) was used as the theoretical framework for two reasons. First, this is one of the first theories on wayfinding concerning the spatial environment. Second, this theory has been applied by other researchers for seniors with dementia.

Passini's theory (Passini, 1984) describes three cognitive steps: (1) processing environmental information, (2) making decisions and development of a plan, and (3) execution of the plan. This is an iterative process, which happens repeatedly while finding your next destination. Within the three cognitive steps, certain wayfinding behaviors are interdependent.

Regarding the first step, “processing environmental information”, the wayfinder first need to consciously perceive the environment by looking at it (wayfinding behavior) (Rainville et al., 2001). This behavior can be identified by gestures on searching (e.g., look ahead or turn the head), facing or reading a specific cue in the physical environment (Mustikawati et al., 2021). In the paragraph on the architectural perspective, physical attributes are further identified.

After processing the environmental information surrounding you, an action plan will be developed (step 2). In order to do so, a cognitive map is constructed. A cognitive map is a mental image of the planned route in space (Hirtle, 2009). However, due to their dementia, people with dementia gradually lose the skill of making a cognitive map (Miniaci and De Leonibus, 2018; Serino et al., 2014). Nonetheless, research showed that people with dementia can still make sub-plans (Passini et al., 1995) and minor decisions (Rainville et al., 2001; Passini et al., 1998) during the wayfinding process. Associated wayfinding behaviors include verbalizing the desired destinations and directions out loud.

After processing the environmental information and developing an action plan to reach a destination, this action plan will be executed (step 3). This step includes wayfinding behaviors such as the route taken, stops on the route, and opening doors. Route patterns from point A to point B of people with dementia were, for example, identified in daycare centers (Hou and Marquardt, 2015) and in living rooms of nursing homes (van Buuren and Mohammadi, 2023). These route patterns can be categorized as direct, wandering, and no movement (Martino-Saltzman et al., 1991; Algase et al., 2007). Mustikawati et al. (2021) studied people's movements and how they behaved during this movement and observed, for example, shifting in direction, responding to visual information, and stopping during the route. The location of stops on the route for people with dementia was a variable in the daycare study of Hou and Marquardt (2015). Their results showed differences in stop-locations for each spatial design of the three studied day care centers: in doorways, the living room, and not a particular place (Hou and Marquardt, 2015). Other associated wayfinding behaviors to this step are opening doors and receiving verbal navigational cues from care professionals or other people.

The architectural perspective of wayfinding focuses on the design of circulation space and composition. The first wayfinding step involves closely observing the physical environment, searching for cues, involving the building's architecture. The architecture of the physical environment is an interplay comprising shape (e.g., proportions, composition, scale), how the separate elements are interrelated, and the construction of it. The interplay of the architecture and the person includes the usage of a space and the experience of that space, and plays an important role in how the architecture is shaped. In wayfinding, the spaces' composition and the circulation space's design play an essential role, making it possible to move from one space to another.

While this architecture can evoke affective responses, the more functional role of architecture is the focus of this study (Karol and Smith, 2019). This is especially relevant for people with dementia because they become more dependent on the physical space. This is the space they literally can perceive and count on, while the mental image of the physical space declines. Following the Environmental Docility Hypothesis, the physical environment should compensate for these decreasing cognitive skills due to dementia (Lawton and Simon, 1968). Besides architectural elements, interior design aspects are important for this target group. In the Evidence Based Design (EBD) approach, research is conducted on functional architecture to support wayfinding skills (e.g., Weisman, 1981) and specifically for people with dementia (e.g., Marquardt and Schmieg, 2009; Netten, 1989; Passini et al., 2000).

Functional architecture could play, for example, a role in the interpretation of space (e.g., visual accessibility, color) and in supporting wayfinders at decision moments (e.g., architectural articulation, decreasing the number of decision moments via composition) (e.g., Wiener and Pazzaglia, 2021). Former studies have shown that wayfinding for people with dementia is indeed influenced by architectural characteristics such as spatial organization, circulation, and visibility (e.g., Marquardt and Schmieg, 2009). Distinguishable spaces with unique characters and avoidance of repetitive elements could support people with dementia in wayfinding (e.g., Netten, 1989; Elmståhl et al., 1997). Furthermore, previous research has shown that vivid colors (Cernin et al., 2003) and contrasting colors (Crow et al., 2003) strengthen wayfinding skills for people with dementia. Other studies focused on the effectiveness of different cues in the physical environment to stimulate wayfinding for people with dementia, for example, signage, memory boxes, and other landmarks (e.g., Cogné et al., 2018; Ilem and Feliciano, 2018; Gibson et al., 2004). With the limitation of relatively small sample sizes (i.e., *n* = 19; Gibson et al., 2004, *n* = 3; Nolan et al., 2001, and *n* = 6; Veldkamp et al., 2008), they observed that participants with dementia used these cues to find their destination.

To summarize, although some research has been conducted on some wayfinding behavioral patterns, a more comprehensive image of these patterns for people with dementia in nursing home corridors is lacking (Davis and Sikorskii, 2020; Kuliga et al., 2021; Mustikawati et al., 2021). At the same time, insights into the usage of nursing home corridors considering wayfinding is essential to designing well-suited nursing homes for people with dementia. A deep understanding of current wayfinding behavior in these spaces and the design of these spaces themselves is therefore essential. Therefore, this study aims to describe wayfinding behavioral patterns of people with advanced stages of dementia just walking in two nursing home corridors.

## 2 Methods

### 2.1 Research design overview

An empirical study with qualitative methods was conducted to study wayfinding behavioral patterns of seniors with dementia within two nursing home corridors. To describe daily wayfinding behavioral patterns of people with (advanced) dementia, the behaviors were studied via post occupation evaluation methods (e.g., Göçer et al., 2018), namely via fly-on-the-wall observation with a behavioral mapping technique. This observation method provides a systematic, unobtrusive manner of data collection; which suits best to collect data on daily behaviors in the natural living environment. The observed behaviors took place in a specific space: nursing home corridors. In addition, this spatial context was studied by manual building analysis and space syntax to determine *where* certain wayfinding behaviors took place and *which* architectural cues were used (e.g., signage, windows) (see “Case studies”), since the spatial design of corridors influences the wayfinding behaviors (e.g., Passini, 1984). In the following paragraphs, detailed information is provided about the data collection and analysis, as well as the case studies and participants.

### 2.2 Fly-on-the-wall observation with behavioral mapping technique

#### 2.2.1 Data collection

Based upon Zeisel (1981), the observation protocol was developed on “*who* is doing *what* with *whom*, in what *relationship, when*, in what *context* and *where*”. The observation protocol consisted of three templates. The first template was a table on paper containing the categories: when, who, what, whom, relationship, and context. For the category “*what*”, a separate list with “actions” was developed based on the three steps of wayfinding (Table 1, see also Section 1) (Passini, 1996). The categories “*who*” and “*whom*” were filled in by using a code per participant (i.e., 1A), visitor (i.e., 1A′), or care professional (i.e., CP). The category “*relationship*” pointed out the initiator of the action and if physical help was provided during that action. The time stamp was noted in the category “*when*” column. In the column “*context*”, additional remarks were noted, for example if a certain door was open or closed.

**Table 1:** Observation list on the category “what”.

**Wayfinding step**	**Variable**	**Description**
Processing environmental information	Looking at…	Looking at information (signage, nametag, picture, landmark), or at visual accessibility (internal window, open door)
Making decisions and development of plan	Destination	Pronounce aloud destination
	Cognitive mapping	Pronounce aloud directions
Execute the plan	Help	Verbal navigational cues from care professional, physical help from care professional
	Usage	Trying to open doors
		Movement	1. Route taken 2. Stops on the route

The second protocol template was a floorplan of the corridor and adjacent spaces. The categories “*context*” and “*where*” were pointed out on these printed floorplans. The observed behavior was linked to the location where it occurred by coding it on a floorplan. For “*context*”, additional information about the physical situation was pointed out on the floorplan, for example if a door was closed or open.

The third protocol template was developed to create an overview of the participants. Per participant, a small table was provided on the categories: personal (i.e., male/female, clothing to recognize the participant), health (i.e., mobility, glasses/hearing devices, impaired motor abilities, medication), and lifestyle (i.e., hobbies). Next to these categories, space was provided to draw an image of the participant.

In preparation for the structured fly-on-the-wall observation, a site visit to both case studies and informal conversations with care professionals were held beforehand. The site visits contributed to the spatial analysis of the case studies. The informal conversations with the care professionals were meant to gain information about the participants, used for the third protocol template, as well as to introduce the researchers to the care professionals for smooth operationalization during the observations.

Furthermore, an additional day per case study was used afterwards to verify some observations with the care professionals (e.g., if wandering behavior was common for a particular participant, or if a door was normally closed or open). This day was also used for informal conversations with participants to gather possible motivations for certain wayfinding behaviors.

Fieldnotes were made of this unstructured information gathering. The results of the structured fly-on-the-wall observation are complemented with quotes, gained from these unstructured conversations.

The structured fly-on-the-wall observation was applied for 5 days in total: 3 days of observation in CS1 (in April 2022), of which 2 working-days in the continuous loop corridor (between 10:30–16:30 h and 9:30–18:30 h) and 1 working-day in the corridor with bedrooms (between 8:10 and 13:10 h), and 2 working-days in CS2 (in June 2022) (between 10:20–17:20 h and 9:20–16:30 h). To exclude coincidences, multiple working-days per case study were chosen.

**Figure 1:**
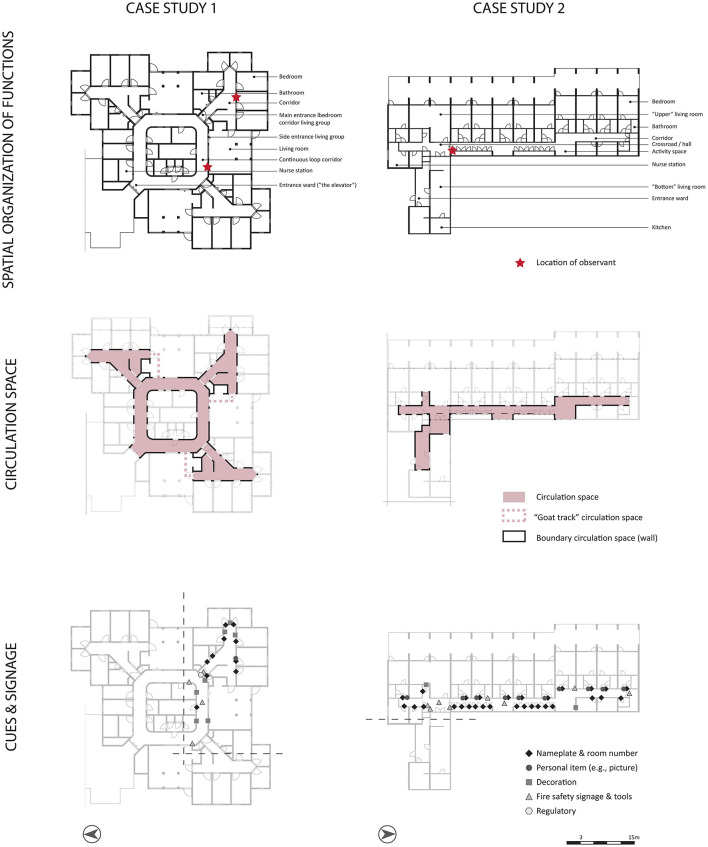
Spatial analysis of the two case studies: spatial organization of functions, circulation space, and type and position of cues.

Each action of the participants was noted during the observational times while the researcher sat on a chair or bench. The position was chosen along the route, instead of at the end of a corridor to avoid being an interesting destination for the participants. Furthermore, from this position, it was possible to oversee most of the corridor, except the behavioral actions behind the corner. In Figure 1, the position of the observer is annotated with a star.

The researcher acted as a recognizable outsider (Zeisel, 1981), meaning that she introduced herself as a researcher but did not participate in the daily routines in the nursing home corridor. In addition, to limit biased behaviors, the researcher did not initiate interaction with the participants during the observations. However, if a participant initiated an interaction (e.g., saying “*hello*”), the researcher responded to this interaction as short as possible to avoid biased behavior.

#### 2.2.2 Data analysis

The handwritten behavioral actions described in the protocol tables (when, who, what, whom, relationship, context) and floorplans (where, context) were converted to written text in Excel and illustrations in Adobe Illustrator on the computer. Thereafter, the category “*what*” based on the variables concerning the three steps of wayfinding (Table 1) was used as the basis for the analysis.

First, the amount of measurements per specific behavioral action (e.g., looking at) was counted per case study. Second, the location of the specific behavioral action (e.g., looking at) was annotated on a floorplan, using the person-centered behavioral mapping technique. A floorplan per behavioral action with its annotated behaviors was made for each participant as well as for all participants per case study together. Quotes from participants obtained via the unstructured conversations were added to the analysis of a specific wayfinding behavior.

Thereafter, patterns were identified on the amount of certain wayfinding behaviors (e.g., looking at) and the location of this behavior in the corridor. Thereafter, pictures of the environment were added to the analysis to gain information about the spatial context. The patterns were discussed with all authors.

### 2.3 Participants and case studies

#### 2.3.1 Case studies

**Figure 2:**
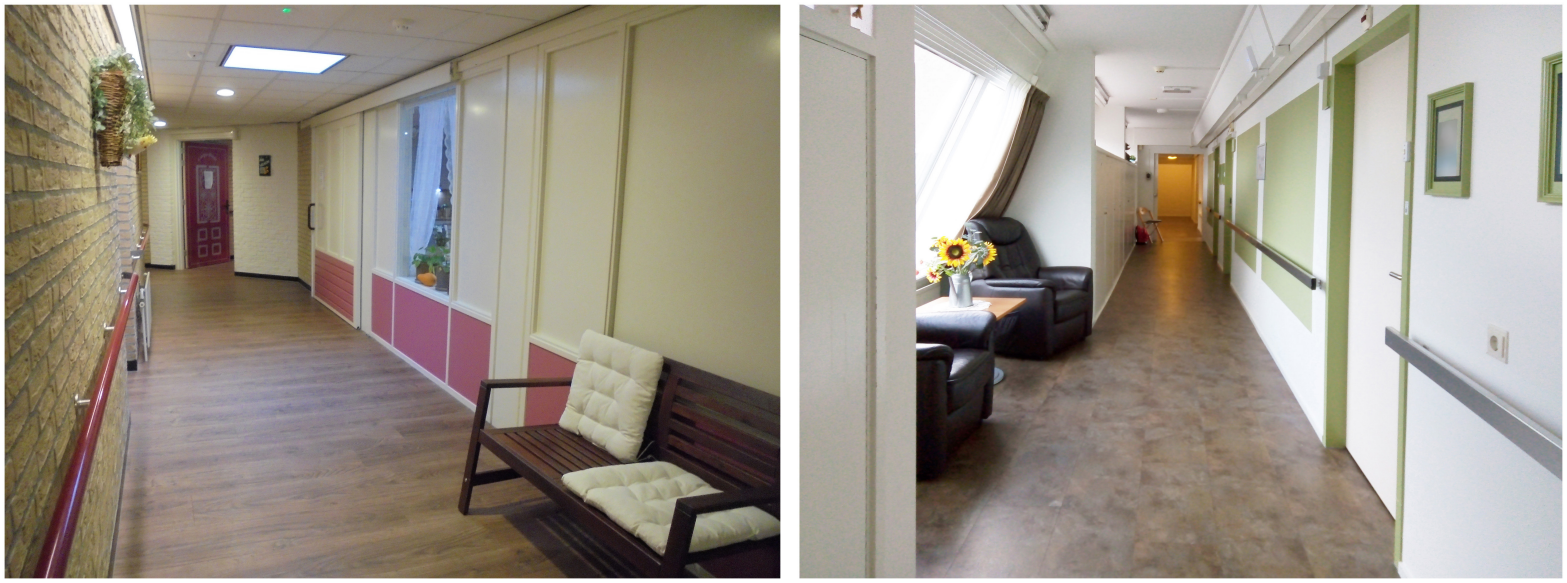
Impression of the continuous loop corridor in CS1 **(left)** and impression of straight corridor in CS2 **(right)**.

The study was carried out in two different nursing homes in the Netherlands (CS1, CS2; see Figure 2 for an impression). Dutch nursing homes typically follow a similar (care-related) organizational structure: a group of people with dementia lives together in a ward with a private bedroom and a shared living room under the supervision of a care organization (Mohammadi et al., 2019; Van Liempd et al., 2009). Spatially, private bedrooms, bathrooms, living rooms, and a kitchen to house the residents are provided. However, how these spaces are arranged and related to each other differs. The circulation space connects the bedrooms with the living room in both case studies. Residents were not allowed to leave the ward unattended. Therefore, the entrances toward the wards were “hidden”, and circulation happened mostly between the bedrooms and the living room(s).

CS1, built in 1989, is situated in a large building complex of two floors with multiple wards (either for people with somatic issues, or for people with dementia). The building also contains facilities for the residents (e.g., restaurant, activity rooms), daycare spaces, a secured garden, and supportive facilities. In total, the nursing home houses 212 residents, and the selected ward houses 18 people with severe dementia. The selected ward is situated on the upper floor. The spatial arrangement of functions of the ward is displayed in Figure 1.

CS1 has two types of corridors: a continuous loop corridor and a straight corridor (van Buuren and Mohammadi, 2022) (Figure 1, circulation space). The continuous loop corridor provides endless walks for residents. From this corridor, the three living groups (via the entrance door and side entrance door), staff supporting spaces, and the entrance/exit to the ward were accessible. This corridor had straight light-beige brick finishes on the walls, white doors for staff supporting facilities, colored entrance doors to the living group, and contrasting red hand rails. The flooring had a wood-like finish and a dropped ceiling with multiple skylights (due to its position on the upper floor) offering some natural daylight. Lighting levels were merely based on artificial lighting. Differentiation in this corridor was limited due to the same materials and colors on the walls, with some minor pastel differences near the living room. Interior windows made it possible to glimpse into the living room from the corridor. The walls were covered with artwork and other decorations. One part of the corridor has a bench to rest; the researcher used this bench during the observation.

The straight corridor in CS1 contained a crossroad: toward the living room, the continuous loop corridor, and the bedrooms. It also had light beige brick finishes on the wall and red handrails. The doors of the individual bedrooms were colored pink, and the other doors in the living group were blue. Also, a wood-like floor finish and a dropped ceiling with a skylight were used offering some natural daylight. Lighting levels were in this corridor also mainly based on artificial lighting. Each bedroom entrance had a nameplate, sometimes accompanied by a picture of the resident or some other artwork. In this corridor, a small activity space was created. This straight corridor is duplicated three times in the ward. All three of them had similar shape, material and color use. Minor changes in the art work at the bedroom entrances provided differentiation between these corridors. The same applied for the three living rooms in the ward: all minor differentiation in pastel colors, but with the same shape and material. Regarding cues, five categories of signage and cues have been applied: nametags and room numbers, personal items (e.g., picture), decoration, fire safety signage and tools, and regulatory signage (Figure 1, signage and cues). The signage is rather small in size with black typography on a white background. Decoration cues are for example a painting or a 3D bird. Only one personal bedroom has both a nametag and personal item (e.g., photograph).

The living room can function as circulation space as well in CS1. Via the side entrance door connecting the continuous loop corridor and the living room, it was possible to access the straight corridor with the bedrooms (Figure 1, circulation space, dotted line).

CS2, built in 1973, is situated in a large building complex with two floors. The building has one closed ward for people with dementia (i.e., the selected ward) and multiple other wings for people with early stages of dementia. In addition, the building houses facilities for the residents (e.g., restaurant), a secured garden, and supportive spaces. In total, the nursing home houses 57 residents, and the selected ward houses ten people with severe dementia. The selected ward is housed at the first floor. The spatial arrangement of functions of the ward is displayed in Figure 1.

CS2 has a floorplan layout system with one straight corridor with a jog (Figure 1, circulation space). This corridor contained dead ends, which residents bumped into on their walks. Bedrooms were situated on one side over the length of the corridor, interrupted by a living room and a bathroom. The other side of the corridor housed staff supporting spaces (including cabinets), rest spaces, and the other living room. It had white painted walls with green squares and chrome handrails. The doors to the bedrooms were also painted white but had contrasting green door frames. In front of the bedrooms, white cabinets were positioned. The corridor had multiple windows providing natural daylight and views outside. The corridor contained repetitive elements (i.e., doors, colored planes, cabinets), but they were interrupted by architectural differentiation in niches. The flooring had dark finishes, and the ceiling was painted white. The doors toward the living rooms had glass in them, allowing residents to look into the living room standing in the corridor. Each bedroom entrance had a nameplate, sometimes accompanied by a picture of the resident. Besides these nametags, room numbers, and personal cues (i.e., picture), cues regarding fire safety and decoration were applied (Figure 1, signage and cues). Decoration was portrayed via paintings and a window sticker of flowers. A cue for the toilet was created by a white A4 paper with black letters and an icon of a man and lady. The corridor contained two activity spaces: one with two lazy armchairs and the other with a bench, bookshelves, and a cradle.

**Figure 3:**
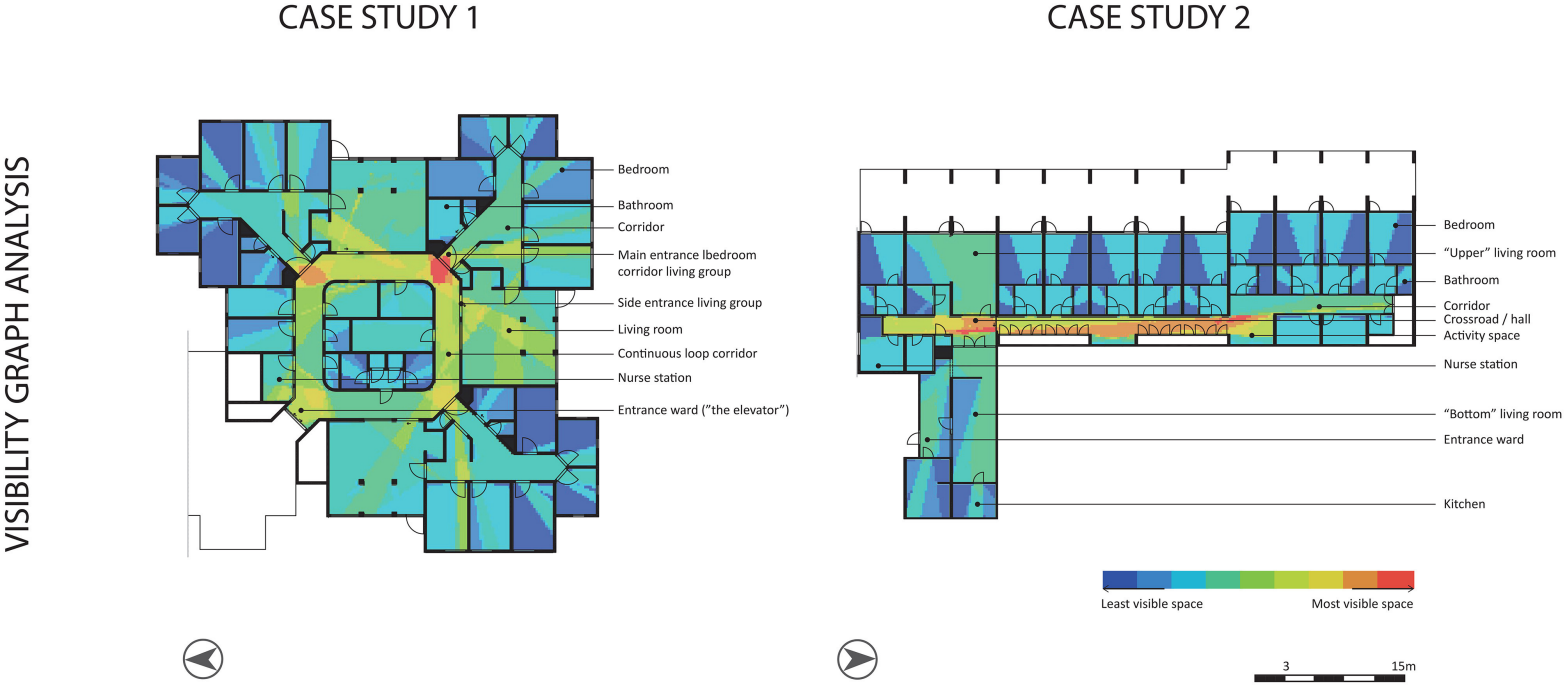
Visibility graph analysis of the two case studies.

A visibility graph analysis (VGA) (software depthMax, a grid of 200, global measures “*n*”) revealed the most and least visible spaces in the corridors (Turner et al., 2001) (Figure 3, VGA). The most visible space in CS1 was situated in the entrance zone of the observed living group (situated on the right) and in CS2 in the hall/crossroad. The entrances to the bedroom corridors of CS1 (corners in the continuous loop corridor) were decently visible and accessible for the residents. In CS2, the end of the corridor at the north and south side of the building is decently visible, but were not accessible for the residents.

#### 2.3.2 Participants

In total, eight seniors with advanced stages of dementia [i.e., Alzheimer's Disease and one senior with frontotemporal dementia (participant 1D)] (*n* = 8) participated in the study, four per location. Only participants who could move autonomously to identify their wayfinding behavioral patterns were included in the study. Participant 1A (case study 1; participant A) had a walker, and participants 2A and 2C needed a walker but often moved without this walker. Participant 1C used a stick for short distances within the nursing home and a wheelchair for longer distances outside the nursing home. Participants 1B, 1D, 2B, and 2D had no aids for mobility. Table 2 shows an overview of participant characteristics concerning aids for mobility, aids for hearing and seeing, and hobbies or preferred activities.

**Table 2:** Characteristics of participants.

		**Aids for mobility**	**Aids for hearing and seeing**	**Hobbies or activities**
Case study 1	A	Walker	Glasses	Very active; lots of activities
	B	n.a.	n.a.	Walks a lot
	C	Stick for short distances within the nursing home ward, wheelchair for longer distances	Glasses and hearing device	Daily partner visit
	D	n.a.	n.a.	Spends most of her time individually
Case study 2	A	Moves often without walker, but actually needs one	Glasses	Personal attention
	B	n.a.	Auditive issues	Doing daily things on her own
	C	Moves often without walker, but actually needs one	Glasses	Small activities at table
		D	n.a.	n.a.	Sporting activities

### 2.4 Research ethics

The participants in our research are due to their condition by definition vulnerable and incapable to communicate their wishes well. Therefore, it is important, even more than in studies involving regular respondents, to protect them and to treat them with most care and respect. This automatically implies that well-thought research ethics are unbearable. In the preparation phase, the study set up was discussed with the data steward and privacy officer of the ethical review board of the Eindhoven University of Technology. This board approved the study (ERB2022ID68a). Collaboration agreements were signed by both care organizations and the Eindhoven University of Technology. Furthermore, meetings were held with different management layers and care professionals of the care organization to inform them about the study. The care professionals who know their residents well, recruited the participants in close collaboration and informed consent of the main responsible informal caregiver. Besides, participation was completely voluntary and there was no pressure to participate. If participants changed their mind about participating that was fine and they could withdraw from the study at any time during the data collection phase.

In the data collection phase, data was collected about the spatial context and the daily wayfinding behavioral patterns of the participants. In taking the photos, we took care to ensure that no respondents were recognizable in the picture and visible nametags or profile pictures were blurred. The daily wayfinding behavioral patterns were only collected for residents who agreed upon participation, for 2–3 days per case study. Participants were not mentioned by names, but by codes (e.g., Participant A). We studied their wayfinding behaviors in their own living environment, implying that the participants were not asked to go elsewhere to collect data. Also, the observant acted as a recognizable outsider, meaning that she introduced herself at the start of the observation and during the observation when asked. This was the least invasive manner of data collection. Furthermore, informed care professionals were present during the data collection phase.

Data was stored on the Research Drive of the Eindhoven University of Technology. This is a storage system where data is safely and securely stored with easy restore and back-up functionality. The system has controlled access and authorization so that only primary team members can access the data. The data will be stored for 4 years after the end of the research project. The original dataset will not be published, but only the results will be published.

## 3 Results and analysis

### 3.1 Wayfinding step 1: processing environmental information

#### 3.1.1 Wayfinding behavior: looking at…

In both case studies, specific participants did observe possible navigational cues in the spatial environment (CS1: 126 measurements, CS2: 164 measurements). These participants showed more movement behavior during the day, except for participant 2D, who could indeed have been searching for navigational cues to lead him back to his spot in the living room departing from the toilet.

**Figure 4:**
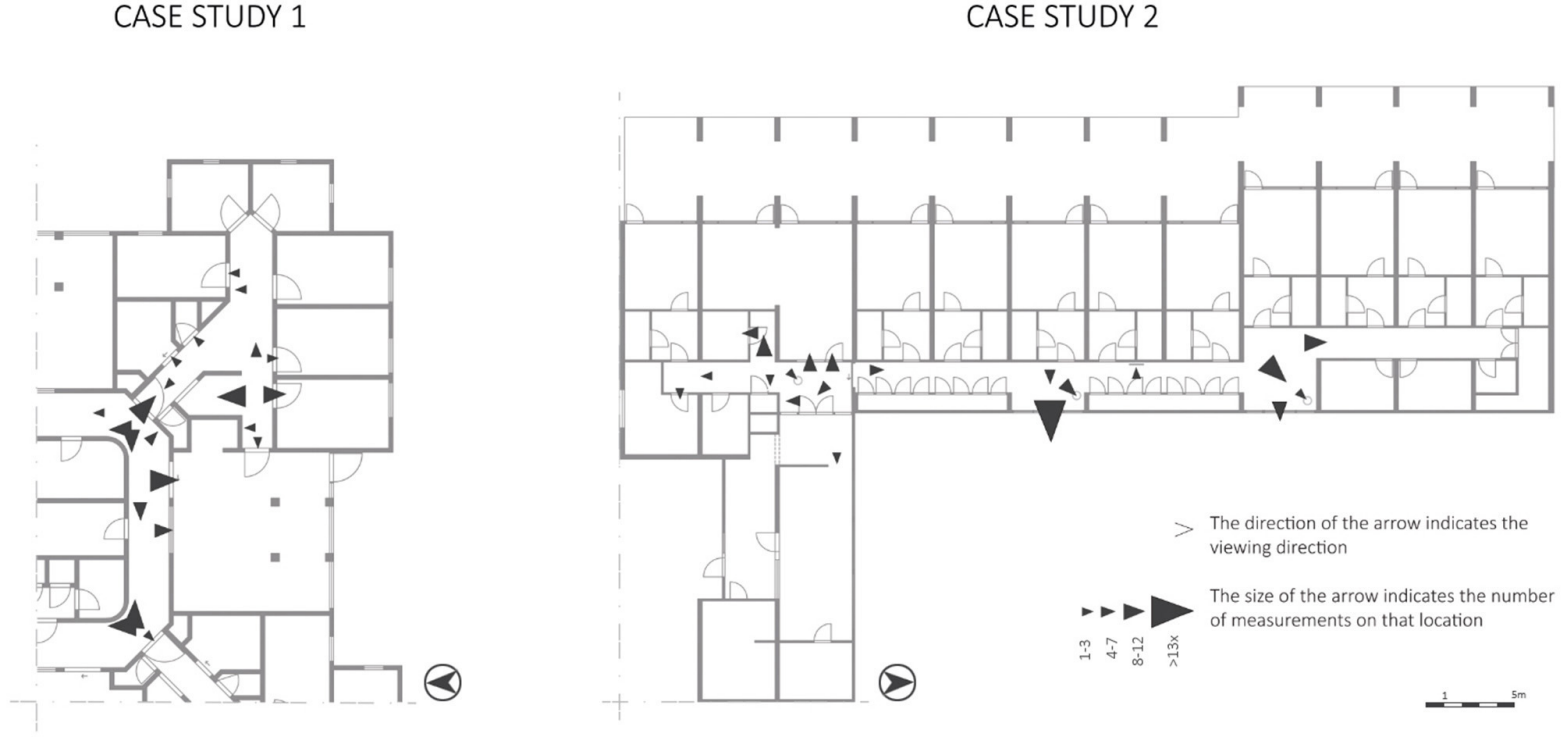
Behavioral actions of the variable “Looking at…” mapped on floorplans.

The possible navigational cues were divided into the following categories: spaces, corridors, windows, objects, and signs. Spontaneous encounters with persons were excluded from this analysis due to the spatial focus of this study. In CS1, the participants mainly looked at corridors (47%) and spaces (41%); while in CS2, the participants looked mostly at windows (29%), spaces (27%), corridors (17%), and signs (16%) (Figure 4). In both case studies, multiple signs and objects were integrated into the spatial environment, but the ones in CS2 were clearer (i.e., larger, more personalized). However, in CS1, a couple of times, participants observed a letter near the entrance door to the living room (i.e., the highest visible space). This letter was meant for visitors instead of the residents.

In both case studies, participants looked through interior windows between the corridor and the living room (5 and 4%). Participant 1B mentioned “*When walking around, I always take a look inside the living room to see if I see someone I like*”, and if 1B did not see someone nice, 1B continued the walk.

In both case studies, participants frequently looked around at crossroads in the floorplan layout; in CS1 at the entrance of the bedroom corridor on the south-east corner (25%), and in CS2 in the hallway in front of the living rooms (17%) (while not all elements were relevant for residents, for example, the fire alarm box or the fire hose). According to the VGA, both places have a high visibility value (Figure 3, VGA). The other most visible place in CS2—the activity space—is also often looked at. In addition, participants often looked at less visible spaces, such as the corridor near the toilet. In CS1, the most frequently looked at spaces are near the side entrance and interior window of the studied living room and the activity space in the bedroom corridor, which is still quite visible.

### 3.2 Wayfinding step 2: making decisions and development of plan

#### 3.2.1 Wayfinding behavior: cognitive mapping and destination

In both cases, only 13 measurements regard pronounced aloud directions (cognitive mapping) and 13 measurements regard pronounced aloud destinations. The majority (77%) of the spoken directions came from participant 2B; the others were from participants 1A, 1B, and 2C. During the observations, participant 2B often spoke aloud during her movements. Examples of directions were: “*This way*” or “*Here we go to the right*” (which was, in fact, to the left).

The destinations mainly were pronounced aloud in CS1 (69%). Participants 1A and 1B pronounced their locations toward each other; for example, “*I have to go to my room to get my wallet*”. Participant 1D pronounced her location to the researcher; to “*get coffee in the living room*”, to “*get towels from the cabinet*”, and to “*go to the toilet*”. Participant 2A pronounced aloud the destination “toilet” to participant 2C; however, participant 2A did not go to the toilet but to the living room.

### 3.3 Wayfinding step 3: execute the plan

#### 3.3.1 Wayfinding behavior: receiving verbal navigational cues

Verbal navigational cues from care professionals to participants were provided only 14 measurements in CS1 and 12 in CS2. Most of these verbal navigational cues (71%) in CS1 were meant for participant 1B. In CS2, these cues were given to all participants.

In CS1, a verbal navigational cue was often provided as “*Come this way*” or “*Look, we will go this way*”. Rarely a final destination (e.g., own room, living room) and never a direction (e.g., left, right) was given. Participants of CS1 were usually physically supported by the care professional by taking his hand or arm or by pointing toward the direction. In CS2, a verbal navigational cue was often given as “*Come this way…*”. Often, the final destination was added to the cue (e.g., living room, toilet, walker), and sometimes a direction was indicated, e.g., “*straight ahead and then to the right*” or “*just straight ahead*”. The participants of CS2 received verbal navigational cues, often without physical help.

**Figure 5:**
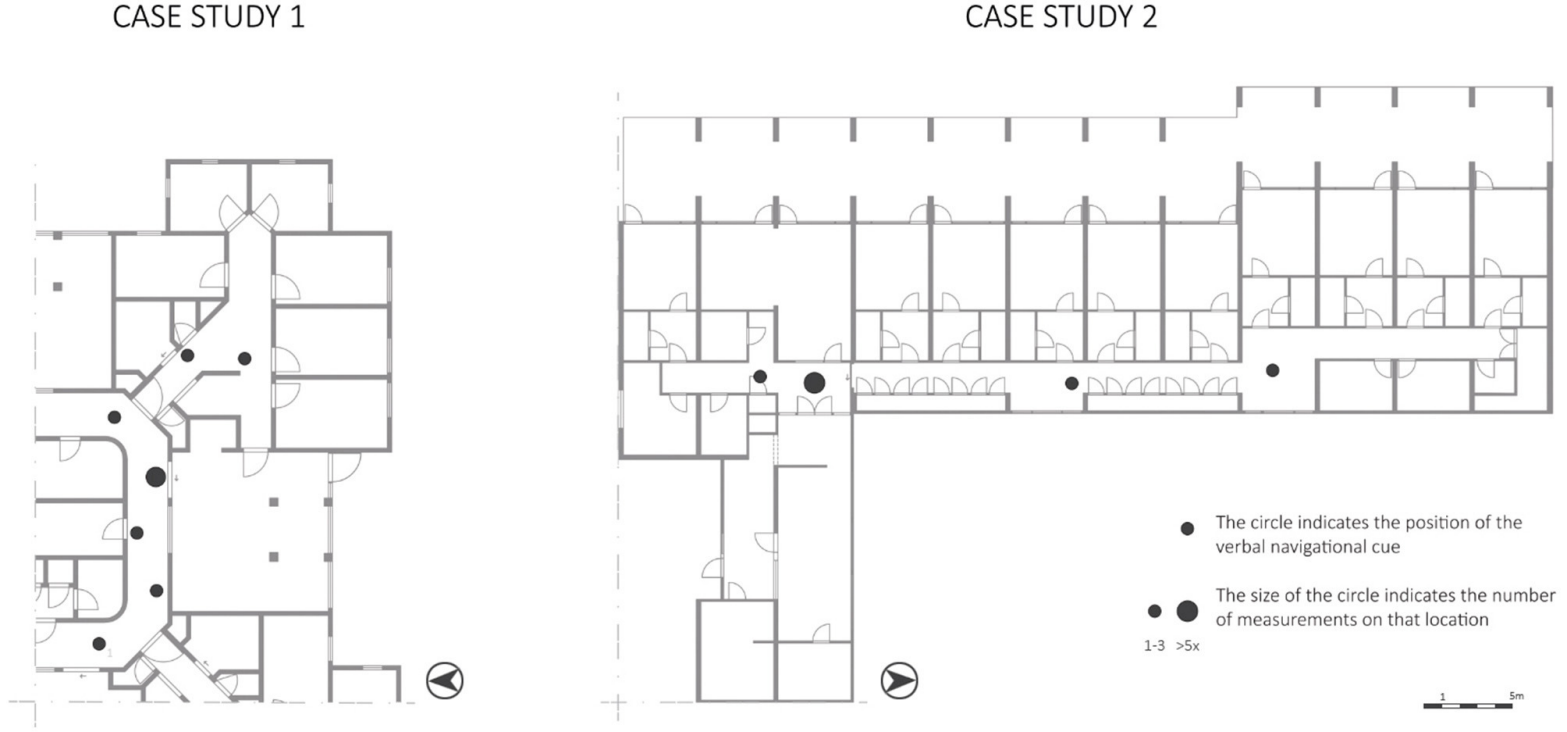
Behavioral actions of the variable “verbal navigational cues” mapped on the floorplans.

The majority of the verbal navigation cues (57%) in CS1 were given around the entrance door at the side of the studied living room (Figure 5). This is a reasonably highly visible area, according to the VGA. Most of the verbal navigational cues (67%) in CS2 were given in front of the entrance to the living room. This is, according to the VGA, the most visible space (Figure 3, VGA).

#### 3.3.2 Wayfinding behavior: opening doors

During the observations, there was a fairly minimal attempt to open doors (CS1: 3 measurements, CS2: 15 measurements). Mainly, participants 1B and 2B tried to open doors. These participants showed more movement behavior during the day. The doors toward the living rooms in both cases were already open for long periods during the day, and the entrance door to the living group of CS1 was open already the entire day.

In CS1, the participant unsuccessfully tried to open the locked doors of the bedrooms of other residents. In CS2, participants unsuccessfully tried to open the locked doors of bedrooms of other residents, successfully opened the door to the toilet, and successfully opened the fire alarm box. Concerning the fire alarm box, participant 2B was reading aloud the text on it: “*Open here*”. After that, participant 2B opened this box and the fire alarm went off.

The entrance door of the bedroom corridor of CS1 is positioned at the most visible place according to the VGA; but was most of the time already open (Figure 3, VGA). In CS2, the fire alarm box is located in the most visible place according to the VGA, which was regularly touched and opened by participant 2B. The door to the toilet was also often touched and successfully tried to open—sometimes without toileting reasons—while this is not a very visible place.

#### 3.3.3 Wayfinding behavior: route taken

As mentioned earlier, destinations of the route were rarely spoken aloud. However, routes taken in the corridors were mapped per participant. This mapping showed that some participants showed frequent movement behavior (1B, 2A, 2B, and 2C). Participant 1A accompanied participant 1B on some walks in the continuous loop corridor in the afternoons. Participant 1D was only seen on the continuous loop corridor on the first observation day. Participant 2D showed more focused movement behavior; from the living room to the toilet. However, sometimes after departure from the toilet, participant 2D searched for his spot in the living room and often walked first to the other living room.

**Figure 6:**
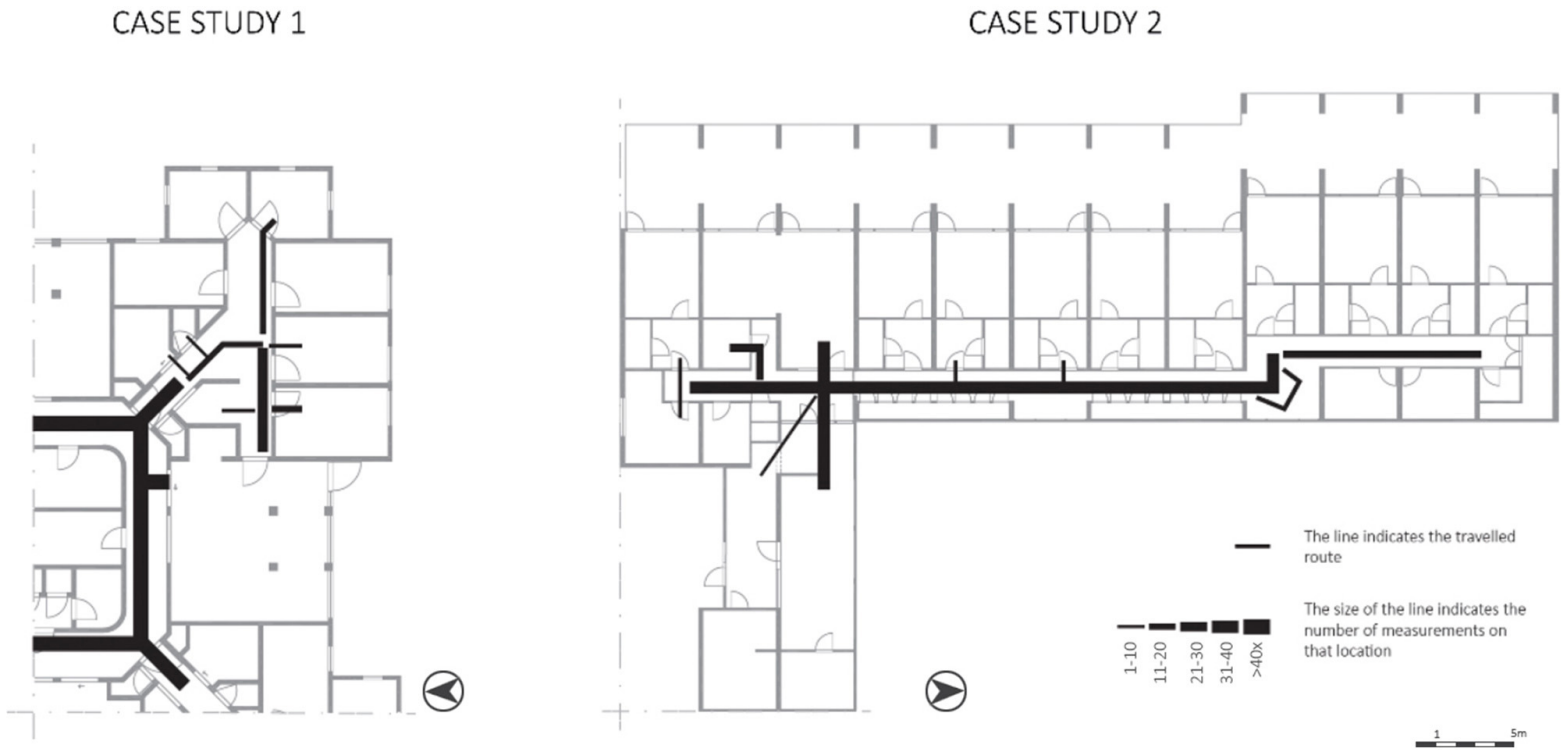
Behavioral actions of the variable “routes taken” mapped on the floorplans.

The entire space the corridors offers, was used by the participants (Figure 6). However, some spaces were used more often than others. In CS1, the continuous loop corridor was used often for routes taken by the participants. In CS2, the hall/crossroad was used more often than the corridor at the right side of the building. This hall/crossroad is a highly visible area according to the VGA (Figure 3, VGA).

#### 3.3.4 Wayfinding behavior: stops on the route

In both cases, particular participants made stops on their routes (CS1: 26 measurements, CS2: 34 measurements). Often, these participants showed more movement behavior.

**Figure 7:**
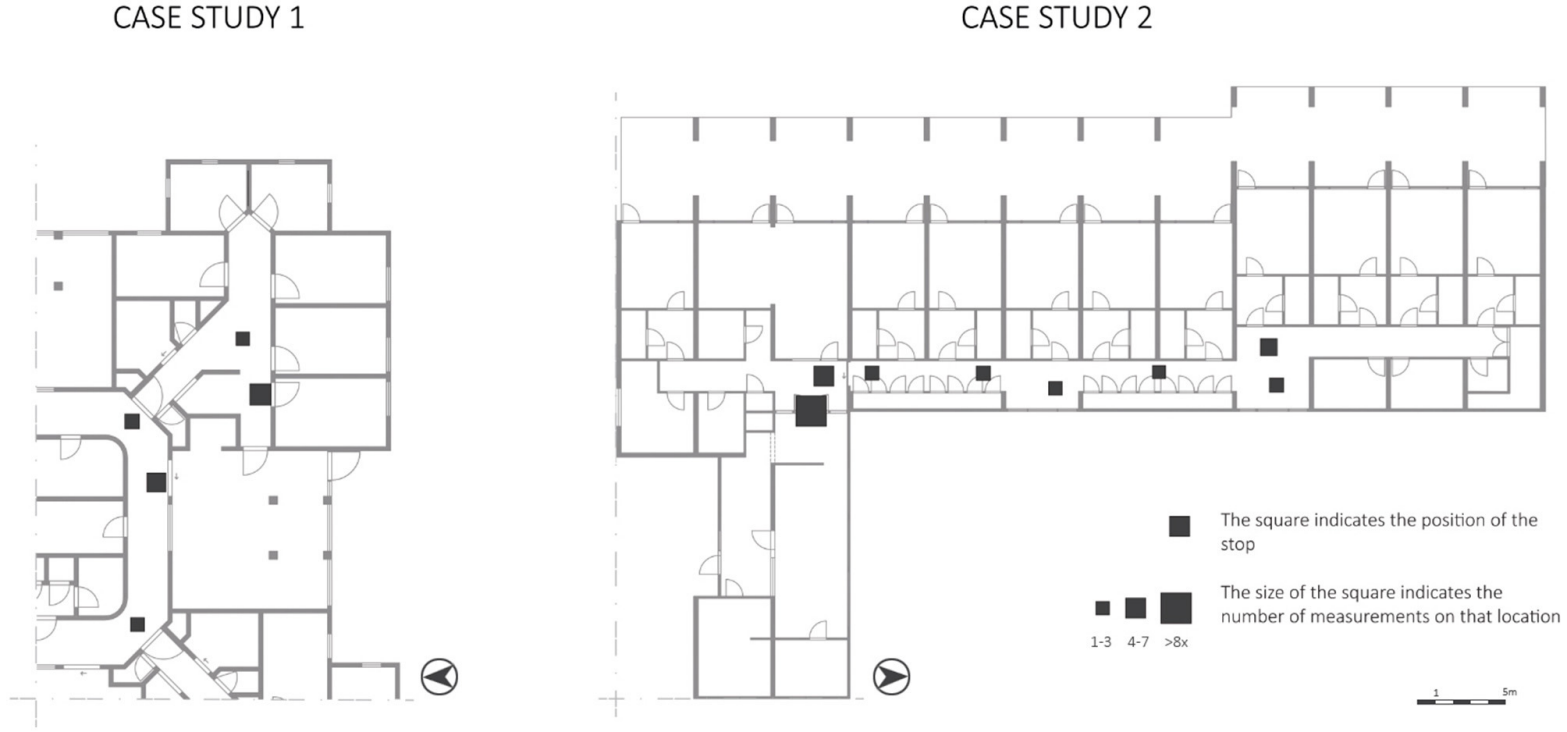
Behavioral actions of the variable “stops on the route” mapped on the floorplans.

In CS1, participants mainly stopped (23%) at the side entrance door toward the living room (Figure 7). This is a reasonably visible area regarding the VGA (Figure 3, VGA). Participant 1B also stopped regularly (31%) in front of a bedroom. This bedroom belongs to the beloved one of participant 1B. In CS2, participants mainly stopped (56%) in the hallway in front of the living rooms; which is also the most visible place according to the VGA.

## 4 Discussion

### 4.1 Observed wayfinding behaviors

This study aimed to describe wayfinding behavioral patterns of people with advanced stages of dementia living in nursing home corridors. Therefore, the movements and related actions of eight people with dementia were observed in two nursing home corridors. Regarding the first step “processing environmental information”, participants indeed looked at corridors, spaces, windows, signs and objects. Previous research already showed that people with dementia used objects, signs, and visual accessibility through interior windows to find their way (e.g., Marquardt and Schmieg, 2009; Gibson et al., 2004). Although some signs in the observed nursing home corridors (i.e., letter for visitors, fire escape sign) and objects (i.e., microwave, care equipment) were not meant for the participants, participants still were observing them. Which was also noted by the research of Davis and Sikorskii (2020). The wayfinding behavioral pattern “looking at” often occurred at decision moments on the route.

Wayfinding behaviors of pronouncing aloud directions (cognitive mapping) and destinations of the second step “Making decisions and development of plan” were observed rarely. This is likely, since few people will verbalize their destinations aloud. However, it still could be the case that participants did this in their minds without pronouncing it out loud. The method of fly-on-the-wall observation cannot be conclusive about this.

The wayfinding behavior “opening doors” of the third step “execute the plan” was rarely observed, although care professionals reported this behavior regularly in daily practice. The doors that were tried to open, were bedroom doors of other residents or the toilet without using the toilet. When it happened, it happened often in clearly visible places in the corridor or at the end of the corridor. The latter was expected because participants approached an endpoint and then wanted to go somewhere.

Although verbal navigational cues are not directly related to the physical environment, the location of this given verbal navigational cue can provide information about the physical environment. It might be that information is missing at the location of this verbal navigational cue to make a choice—or even that there is too much information to decide—and that is why a care professional comes to the resident's aid. Relating the observations of verbal navigational cues to the spatial context, they also occurred often at decision moments. Making decisions becomes harder for people with dementia (Rainville et al., 2001), perhaps clarifying why verbal navigational cues are needed here.

Concerning the “routes taken”, the participants used the available walking space completely.

The stops on the routes taken also occurred often at decision moments. It seems that participants took some time to decide to go left, right, or straight ahead. In CS2, participants also stopped on the route to look outside the window. However, since CS1 had no windows in the corridor to the outside, looking outside was not observed.

Our study revealed that the most observed wayfinding behavioral patterns in both case studies were the “routes taken” (wayfinding step 3), followed by “looking at” (wayfinding step 1), “stops on the route”, and “verbal navigational cues” (both wayfinding step 3). On the other hand, the wayfinding behavioral patterns of “opening doors” (wayfinding step 3), pronouncing out loud “directions” and “destinations” (both wayfinding step 2) were observed rarely.

### 4.2 Personal motivational factors

Some participants, who frequently walked without a specific purpose in both case studies, exhibited behaviors such as “looking at”, “making stops along the route”, and “open doors”. In contrast, other participants or residents rarely exhibited these behaviors. While unstructured interviews provided sometimes information about the motivations, the majority of the motivations still needs to be discovered. Although, this information might be essential to understand *how* people find their way. Based upon this study, we cannot conclude if “unnecessary” objects (e.g., letters meant for visitors, fire alarm box, microwave) should be eliminated from the nursing homes, because we do not know if these objects confused people in wayfinding. Perhaps the “frequent” walkers who often looked at these objects were searching for stimuli rather than destinations on their walks.

Besides the influences of spatial design and the social environment, prior research revealed that emotions also influence the wayfinding process. For example, emotion could influence the acquisition of spatial information or the spatial memory process (Ruotolo et al., 2019), decision making, attention, and working memory (Balaban et al., 2017). Therefore, future research could focus on measuring affective responses of nursing home residents, to gain a deeper understanding of the wayfinding behavioral patterns and its related motivations, as well as if people were conscious about being lost.

### 4.3 Architectural influential factors

In this article, we briefly touched on (the role of) the architecture of the nursing home corridors concerning wayfinding for people with dementia, but we have not fathom it. When considering the present knowledge of EBD (some of which have been discussed in the theoretical background) and evaluating the application of that knowledge in the two case studies, room for improvement can be identified. For example, guidelines concerning signage state that this should be available at eye-level and in an organized manner (e.g., Davis and Sikorskii, 2020; Gross et al., 2004; Wang and Lu, 2022). However, in both case studies, many things are visible in the corridors, e.g., colors of the doors, information board, name tags, greenery, handrail, signals for fire safety reasons, paintings, and furniture. The observations showed that participants did notice these elements. However, the question arises if it is still possible to distinguish potential helpful reference points for wayfinding due to the multitude of colors and stimuli.

Furthermore, differentiation in architecture and interior design is important (e.g., Netten, 1989; Elmståhl et al., 1997). In CS1, the spatial composition of the living group with six bedrooms is duplicated three times, connected by the continuous loop corridor. This results in identical shape and composition. Given the minor interior differences in color, use of materials, and objects, it might not be surprising that people with dementia experience difficulties in finding their way. Also, in CS1, consistency is lacking in the naming of the living groups (based on the names of colors) and the use of color in that particular living room. For example, in living room “Green”, the furniture is colored red; and the front door of living group “Yellow” is painted blue. This might be confusing when colors are intended to guide people. Informal conversations with caretakers made clear that they were unaware of this situation. A strong advice to professionals who deal with the design of the nursing home is to pay close attention to the design and use the existing EBD knowledge. Also, care professionals should be informed about this design characteristics, so they can communicate more suitably on wayfinding purposes for people with dementia. Because, it is important to remember that people with dementia are primarily dependent on the physical environment to guide them.

### 4.4 Visual accessibility and wayfinding behaviors

**Figure 8:**
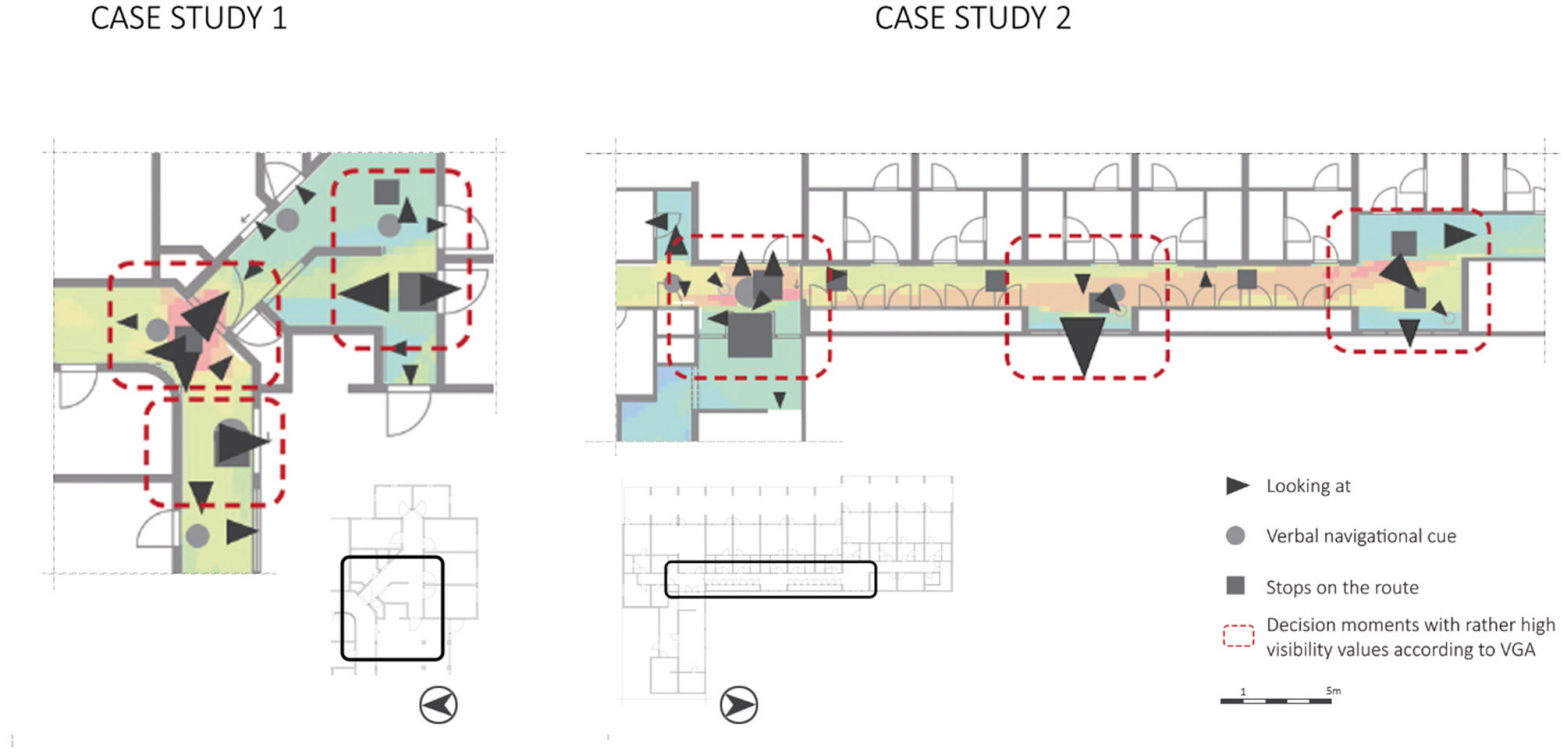
Wayfinding behaviors “looking at…”, “verbal navigational cues”, and “stops on the route” plotted on the VGA of the two floorplans, including marked decision moments.

The space syntax analysis showed places with high visibility values (Figure 3, colored in red) in the two case studies. These spaces are situated at crossroads, which are decision moments for people with dementia. At these crossroads, many things in the building are visible, both architectural (e.g., different corridors, interior windows) and interior (e.g., signs, objects). Considering the observed wayfinding behaviors to these places, this study showed that the behaviors “looking at”, “verbal navigational cues”, and “stops on the route” frequently occurred (Figure 8). Future research is needed to determine whether this is the case or whether it was specific to this sample or type of nursing home. This observation might indicate that places with a high visibility value could confuse people with dementia during their wayfinding activity.

This preliminary finding would contradict earlier research. Research by Li and Klippel (2012) showed that for library visitors, VGA results of space syntax can be used to predict “errors” in the wayfinding process. They found that low-visibility areas led to more errors and mistakes compared to high-visibility areas. Kuliga et al. (2019) revealed similar results in their study in libraries; indicating that places with high visibility areas tend to be anchor points in the building and these places were preferred. Our study provides initial evidence for the opposite; particularly in high-visibility areas, much confusion arose. This raises the question of whether the interpretation of space syntax results differs for people with dementia compared to people with cognitive healthy abilities. A more detailed architectural analysis of these types of spaces, could provide insights into the complexity of these spaces as well.

Visual accessibility—visibility—has been shown in previous research to be important for wayfinding (Weisman, 1981) for people with dementia (e.g., Netten, 1989). However, physical and cognitive accessibility are two different things. Physically being enabled to see possible destinations from your position does not necessarily mean that people with dementia cognitively understand what they see. The question arises of how architecture can cope with this phenomenon.

In addition, if the participants seem to behave more confused at these decision moments, do they also experience stress in these spaces? Or do they feel relaxed stop on the route, look around, and get help from a care professional at these decision moments? Future research could look into this aspect to gain more insights into the experience of people with dementia at decision moments. This could provide new insights for the design of nursing homes.

## 5 Conclusion

This research focused on observable wayfinding behavioral patterns in nursing homes of people “just” walking in the space. Two important conclusions can be drawn. First, the behaviors “looking at” (wayfinding step 1), “routes taken”, “stops on the route”, and “verbal navigational cues” (wayfinding step 3) occurred most and were exhibited by people who moved frequently. To the best of our knowledge, this information about people with dementia in nursing homes was so far unknown. Second, we observed that these wayfinding behaviors occurred mainly at decision moments. Stops on the route could indicate that people with dementia take time to decide at these crossroads, that they look around for (recognizable) cues in the spatial environment which could help them in continuing their route in the right direction, and that care professionals providing verbal navigational cues support the people. In addition, the VGA showed high values of visibility at these decision moments. While previous research mentioned that high-visibility areas should facilitate wayfinding for people with dementia, the current study showed some initial evidence that these high-visibility areas could also confuse people with dementia during wayfinding.

One of the limitations of this study is the relatively small sample size, which has implications for the generalizability of our findings. However, it is important to keep in mind that the population of older adults with dementia is highly heterogeneous, encompassing considerable individual variation in cognitive and physical health status, environmental influences, and other factors (Cohen-Mansfield, 2000). As such, increasing the sample size may not directly enhance the reliability of our findings, as it could also introduce more error variance due to the diverse nature of the sample. Additionally, recruiting participants from this vulnerable group presents unique challenges; informed consent is required from both caregivers and family members, adding a layer of complexity to participant recruitment.

Furthermore, this study delved into wayfinding behaviors of people “just” walking in space. They were not actively engaged in wayfinding tasks by the researchers, and therefore, we cannot infer with any accuracy if the resident was trying to reach a predefined destination, or was just walking around without a destination in mind. In future research, the wayfinding behaviors of people with dementia should also be studied during a navigation task.

Future research could dive into the experiences of people with dementia at decision moments and the architectural characteristics of these decision moments. The combination of behavior, experience (e.g., affective state, emotional experience), and environmental characteristics could guide architects in designing facilitating architecture for people with dementia while wayfinding in nursing home corridors.

## Data Availability

The raw pseudonymized data supporting the conclusions of this article will be made available by the authors on request.
